# Supplementary material for: Methanogenesis in oxygenated soils is a substantial fraction of wetland methane emissions
Source: Nat Commun. 2017 Nov 16;8:1567. doi: 10.1038/s41467-017-01753-4 (PMC5691036; doi:10.1038/s41467-017-01753-4)
Supplement: Supplementary file 1 — Supplementary Information [file 41467_2017_1753_MOESM1_ESM.pdf]

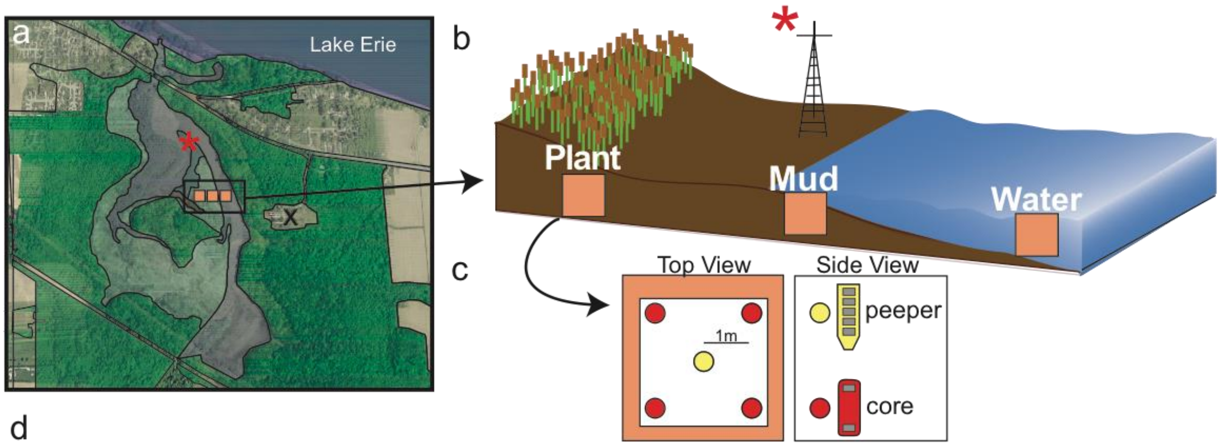

d

Season	F	F	F	F	F	F	S	S	S	S	S	S	
**Year**	2014	2014	2014	2014	2014	2014	2015	2015	2015	2015	2015	2015	
**Ecosite**	P	P	M	M	W	W	P	P	M	M	W	W	
**Depth**	S	D	S	D	S	D	S	D	S	D	S	D	**Sum:**
methane flux	2		2		2		2		2		2		**12**
peepers							1	1	1	1	1	1	**6**
core geochemistry*	3	3	3	3	3	3	3	3	3	3	3	3	**36**
core 16S rRNA gene	3	3	3	3	3	3	3	3	3	3	3	3	**36**
core 16S and mcrA RNA	3	3	3	3	3	3	3	3	3	3	3	3	**36**
core metagenomics	1		1		1		1		1		1		**6**
core metatranscriptomics	3		3				3		3				**12**

Supplementary Figure 1 | **OWC Schematic and Sampling Guide Overview**. **A.** The National Oceanic and Atmospheric Administration (NOAA) field site, Old Woman Creek (OWC), is a 573-acre wetland located adjacent to Lake Erie. Orange boxes designate the location of the sampling transect, comprised of soils beneath three ecosites (plant, mud, and water). Here we monitored the biogeochemistry from this transect over two seasons, Fall (November, 2014) and Summer (August, 2015). Previously, we monitored with 16S rRNA gene and geochemistry multiple transects across the site and showed strong replication between cores from the same ecosite[1]. **B.** Inset cartoon depicts the sampling transect in greater detail, showing the 3 ecosites (2 m^2) as well as the meteorological station and eddy covariance tower (indicated by the red star). **C**. Inset of the replicate core sampling within a given transect, showing ~4 soil cores being collected and the corresponding dialysis peeper always located < 1 m proximity to sample cores. Chamber measurements were collected in duplicate over each ecosite as discussed in methods. **D**. A sampling guide including the number of the spatial and temporal sampling events, the total number of samples collected. Note, due to increased *mcrA* qPCR transcript abundance (Fig. 2), metatranscript data collection was performed on plant and mud ecosite samples. Abbreviations used include: ecosite as Plant (P, green), Mud (M, orange), and Open water (W, blue) and soil sample depth as surface (S, 0-5 cm) and deep (D, 25-35 cm).

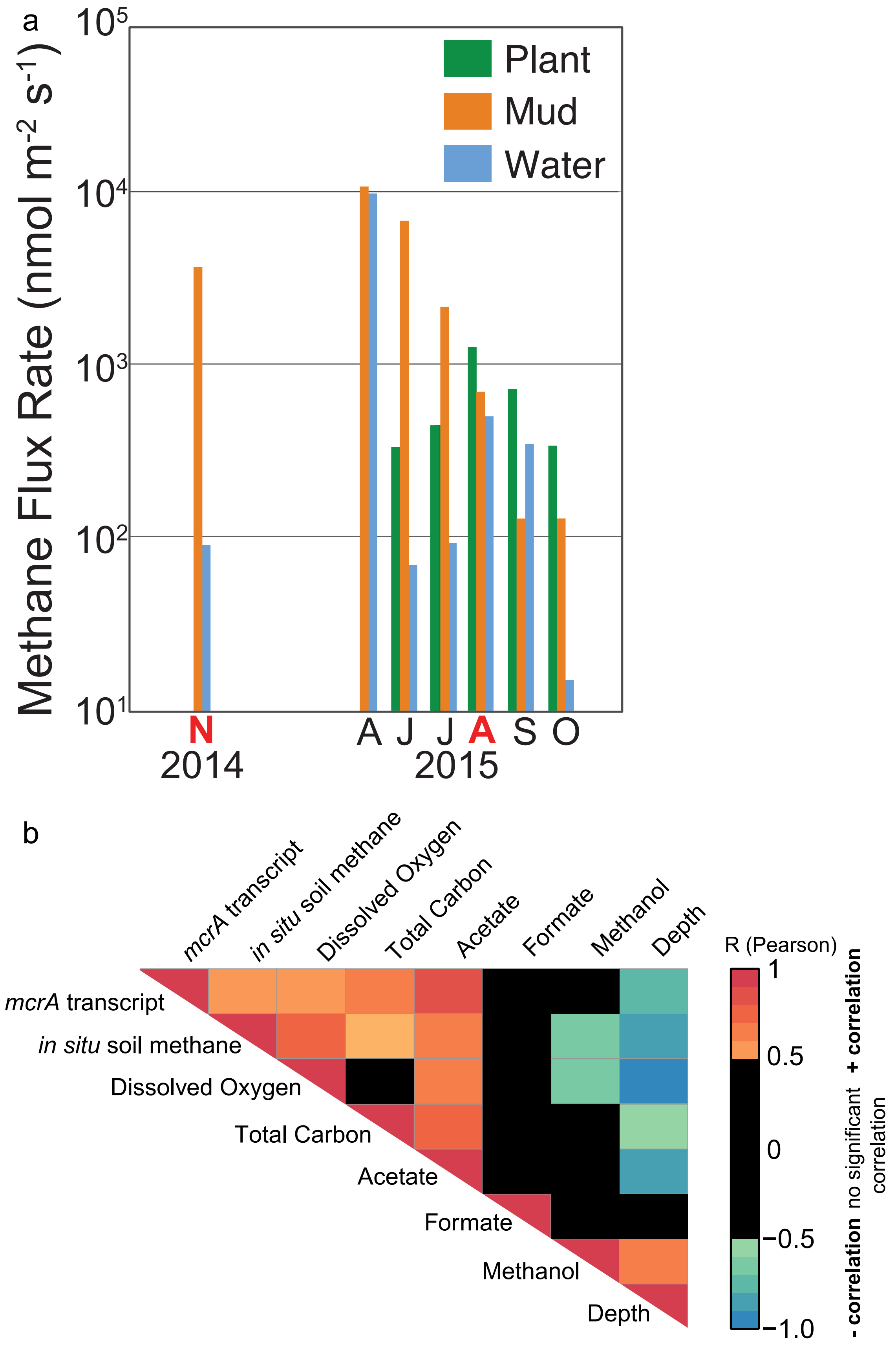
a
Methane Flux Rate (nmol m-2 s-1)
10^5
10^4
10^3
10^2
10^1
Plant
Mud
Water
N
2014
A J J A S O
2015
b
mcrA transcript
in situ soil methane
Dissolved Oxygen
Total Carbon
Acetate
Formate
Methanol
Depth
R (Pearson)
1
0.5
0
−0.5
−1.0
+ correlation
no significant correlation
- correlation

Supplementary Figure 2 | **Methane emission rates and correlation of methanogenic activity to geochemical parameters. A.** Methane flux was measured via non-steady state chamber method, while paired emission, biological and geochemical samples were collected in November 2014 (N, red) and August 2015 (A, red). The x-axis depicts the chamber samplings across time, with each time point consisting of monthly flux data from the ecosites represented by color. Positive methane flux rate is depicted on the y-axis. **B**. Summer soil methanogenic activity (from qPCR of *mcrA*) and corresponding soil geochemistry measurements were assessed for significant correlations. Surface and deep soil sample data from triplicate cores (3 each from plant, mud, and open ecosites) is included in the analysis (Supplementary Data 1). The heatmap depicts Pearson correlation where statistically significantly ($p<0.05$) positive correlations (orange/red), statistically significantly negative correlations (green/blue), and a lack of a significant correlation (black). The correlation between *mcrA* transcript number and acetate concentrations, suggests an important role for acetoclastic methanogenesis in these wetland soils, findings consistent with other reports from soils and lakes[2]. Data regarding the ecosite-level differences in methane emissions have been discussed previously[3].

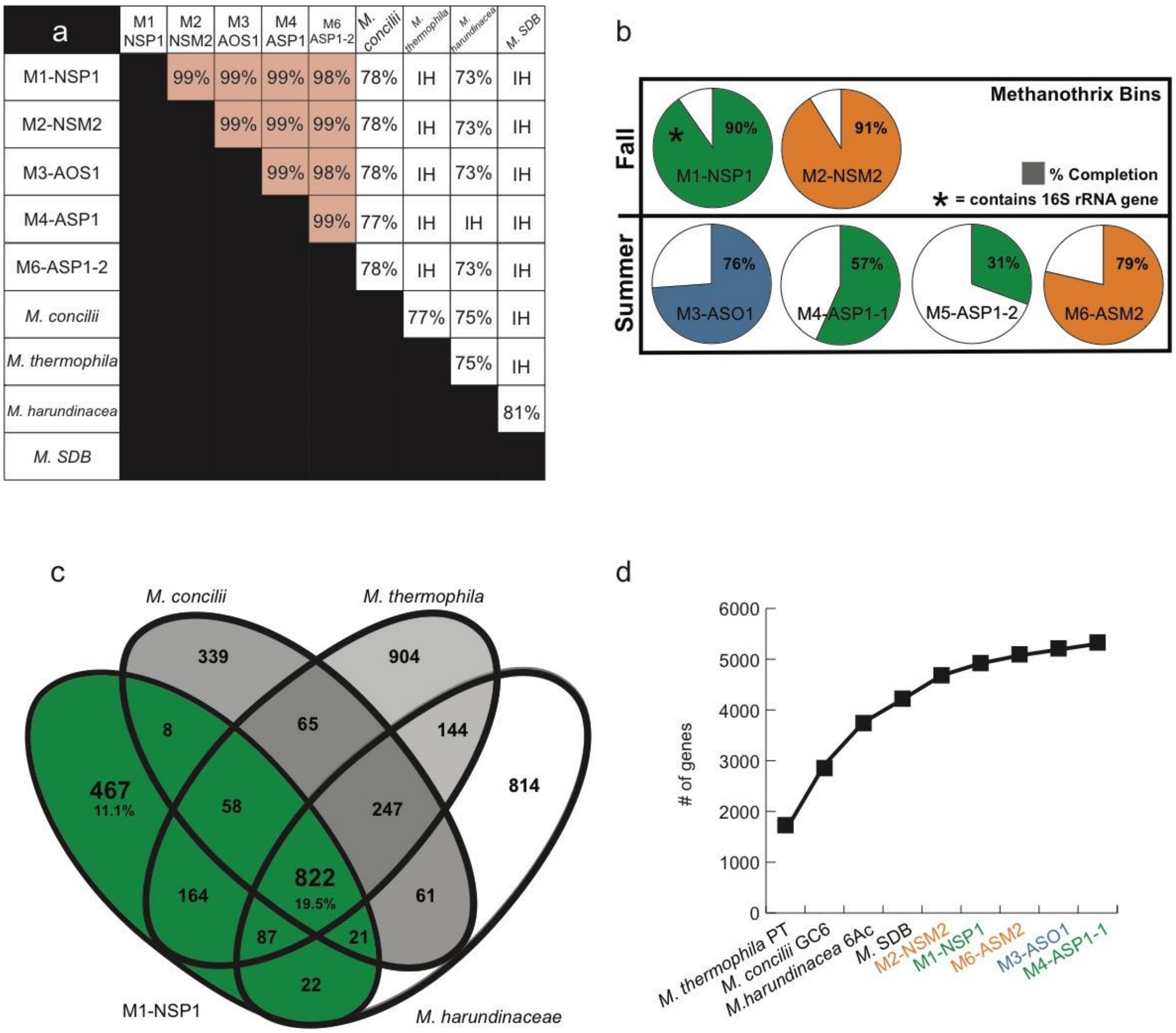

Supplementary Figure 3 | Genome recovery and **average nucleotide identitity reveal a new species of *Methanothrix* termed Ca. Methanothrix paradoxum**. **A.** Similarity matrix of average nucleotide identity (ANI) between reconstructed *Ca.* M. paradoxum genomes greater than 50% complete (M1-M4, M6) and other available *Methanotrix* genomes. **B**. Pie-chart representation of recovered *Ca.* M. paradoxum genome completeness, based on single copy gene analyses, coloring denotes ecosite source with orange (mud), green (plant), blue (water). **C.** Comparative genome analyses revealed flexible and core *Methanothrix* genomes, with 467 genes unique to *Ca*. Methanothrix paradoxum genome. **D.** Pan-genome analyses demonstrated the contribution of each *Methanothrix* genomes (>50% complete) to the total pan-genome of *Methanothrix* genus.

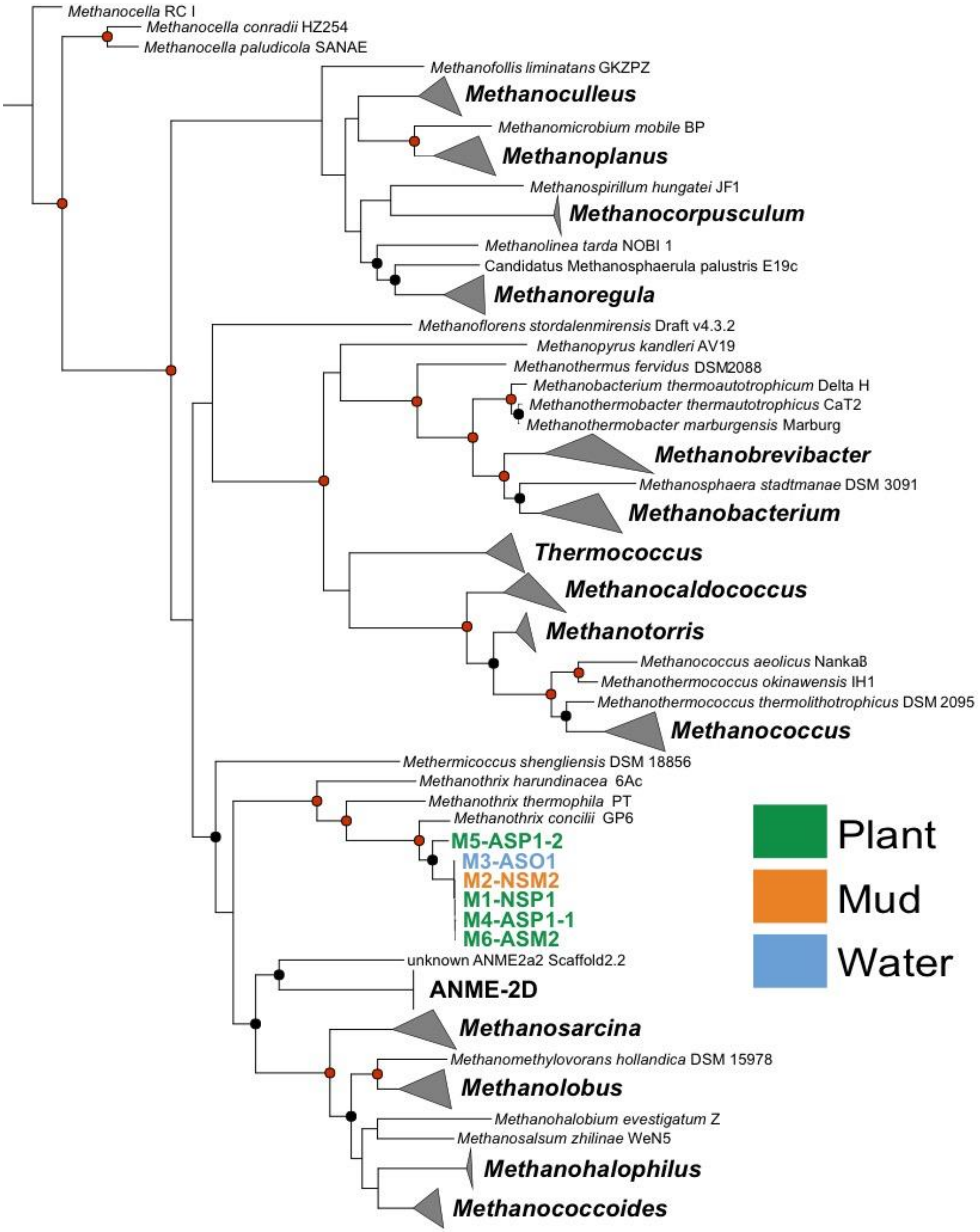
Methanocella RC I
Methanocella conradii HZ254
Methanocella paludicola SANAE
Methanofollis liminatans GKZPZ
Methanoculleus
Methanomicrobium mobile BP
Methanoplanus
Methanospirillum hungatei JF1
Methanocorpusculum
Methanolinea tarda NOBI 1
Candidatus Methanosphaerula palustris E19c
Methanoregula
Methanoflorens stordalenmirensis Draft v4.3.2
Methanopyrus kandleri AV19
Methanothermus fervidus DSM2088
Methanobacterium thermoautotrophicum Delta H
Methanothermobacter thermautotrophicus CaT2
Methanothermobacter marburgensis Marburg
Methanobrevibacter
Methanosphaera stadtmanae DSM 3091
Methanobacterium
Thermococcus
Methanocaldococcus
Methanotorris
Methanococcus aeolicus Nankaß
Methanothermococcus okinawensis IH1
Methanothermococcus thermolithotrophicus DSM 2095
Methanococcus
Methermicoccus shengliensis DSM 18856
Methanothrix harundinacea 6Ac
Methanothrix thermophila PT
Methanothrix concilii GP6
M5-ASP1-2
M3-ASO1
M2-NSM2
M1-NSP1
M4-ASP1-1
M6-ASM2
unknown ANME2a2 Scaffold2.2
ANME-2D
Methanosarcina
Methanomethylovorans hollandica DSM 15978
Methanolobus
Methanohalobium evestigatum Z
Methanosalsum zhilinae WeN5
Methanohalophilus
Methanococcoides
Plant
Mud
Water

Supplementary Figure 4 | **A concatenated ribosomal tree depicting the phylogenetic placement of the 6 surface soil-acquired *Candidatus* Methanothrix paradoxum genomes**. 15 single copy ribosomal gene proteins (RpL2, 3, 4, 5, 6, 8, 14, 15, 18, 22, and 24 and RpS 3, 10, 17, and 19) were extracted from the genomes of all *Methanosarcinales* isolate genomes on the Joint Genome Institute-Integrated Microbial Genomes and Microbiomes JGI-IMG/M database (accessed 12/01/16). Bootstrap percentages ≥75 (black) or 100 (red) are denoted by node circle color. All of the *Methanothrix* genomes reconstructed in this wetland (colored by ecosite) are closely related to each other and phylogenetically distinct from previously sampled *Methanothrix* isolate genomes from a thermophilic anaerobic bioreactor, sewage sludge, and anaerobic sludge blanket reactor (Data from IMG). Our wetland genomes (labeled *Methanothrix* 1-6, or M1-M6) represent the first *Methanothrix* genomes reconstructed environmental shotgun sequencing data. These genomes share > 98% average nucleotide identity with each other and < 80% average nucleotide identity with any prior *Methanothrix* isolate genomes, supporting our conclusion that these genomes represent a new *Methanothrix* species, here proposed as *Candidatus* Methanothrix paradoxum. The aligned concatenated FASTA input file used to generate this figure is provided (Supplementary Data 6).

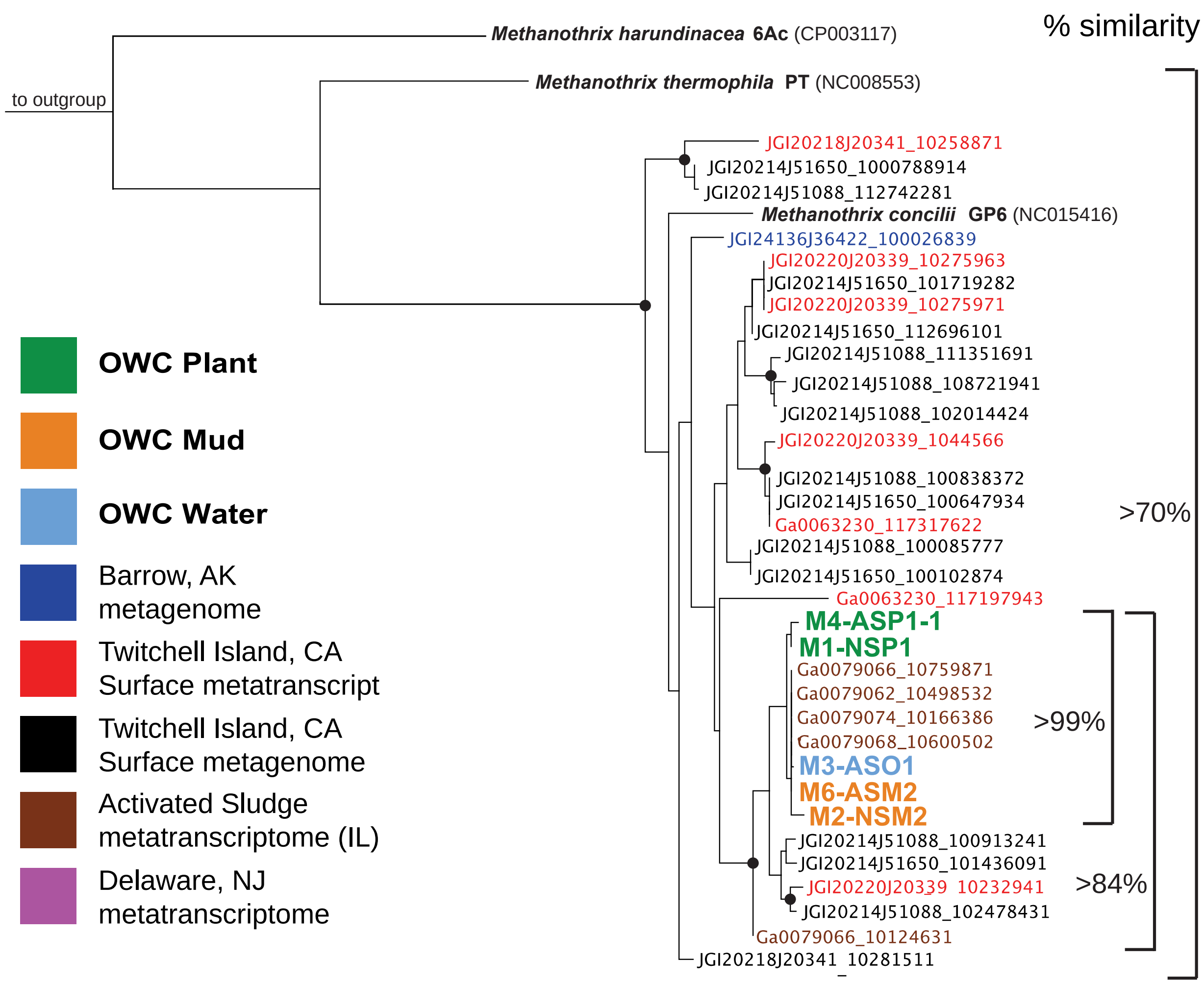

Supplementary Figure 5 | **Evidence that *Candidatus* Methanothrix paradoxum are similar to genotypes in other environmental metagenomes and metatranscriptomes.** We selected the S3 ribosomal proteins (rpsC gene) as a marker as it was consistently transcribed at a high level between summer and fall seasons (Fig. 3C) and in both ecosites. All genes were on unbinned scaffolds, not from reconstructed genomes from environmental metagenomic studies. Ribosomal protein S3 sequences from *Candidatus* Methanothrix paradoxum genome bins are denoted in bold (ecosite denoted by color), while sequences (>70% amino acid identity) recruited from other publically available metagenomic datasets are colored according to the legend. Similar to our findings, genotypes of *Candidatus* Methanothrix paradoxum are active in surface soils from a temperate wetland on Twitchell Island[4] (red and black) and also in activated sludge (brown). Black circles indicate bootstrap values ≥75. The input FASTA file used to generate this figure is provided (Supplementary Data 7).

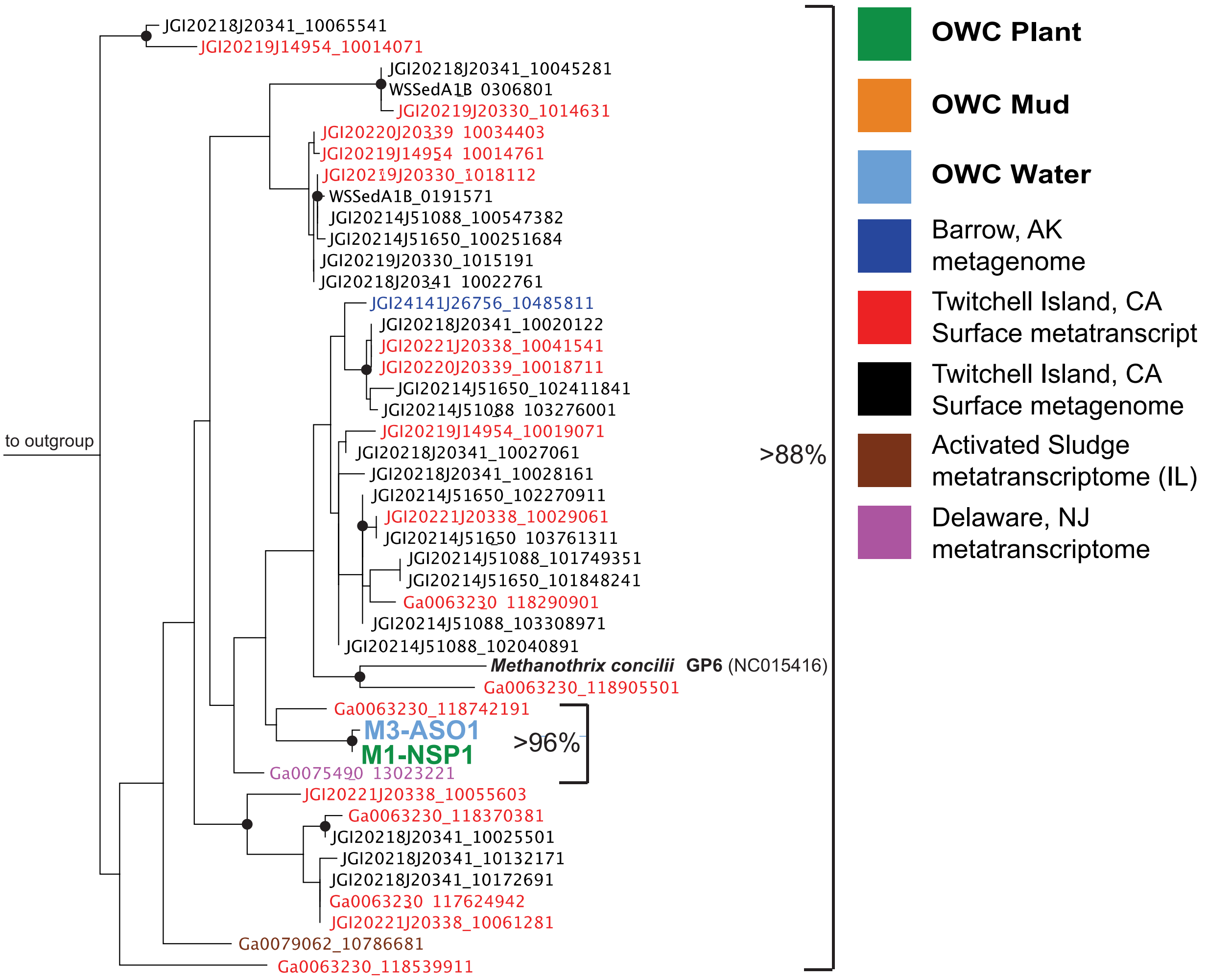

Supplementary Figure 6 | **Evidence that *Candidatus* Methanothrix paradoxum are similar to genotypes in other environmental metagenomes and metatranscriptomes.** Phylogenetic analysis constructed using the methanogenesis functional marker protein *mcrA* amino acid sequences >88% similar to *Candidatus* Methanothrix paradoxum. Binned wetland *Candidatus* Methanothrix paradoxum sequences (bold, M1, M3) are highly similar to transcripts from other surface wetland soils from Twitchell island[4] (red). Sequences included in this analysis had an amino acid similarity ≥88% to sequences from metagenomic datasets on JGI IMG (12/01/16). Black circles indicate bootstrap values ≥75. The input FASTA file used to generate this figure is provided (Supplementary Data 8).

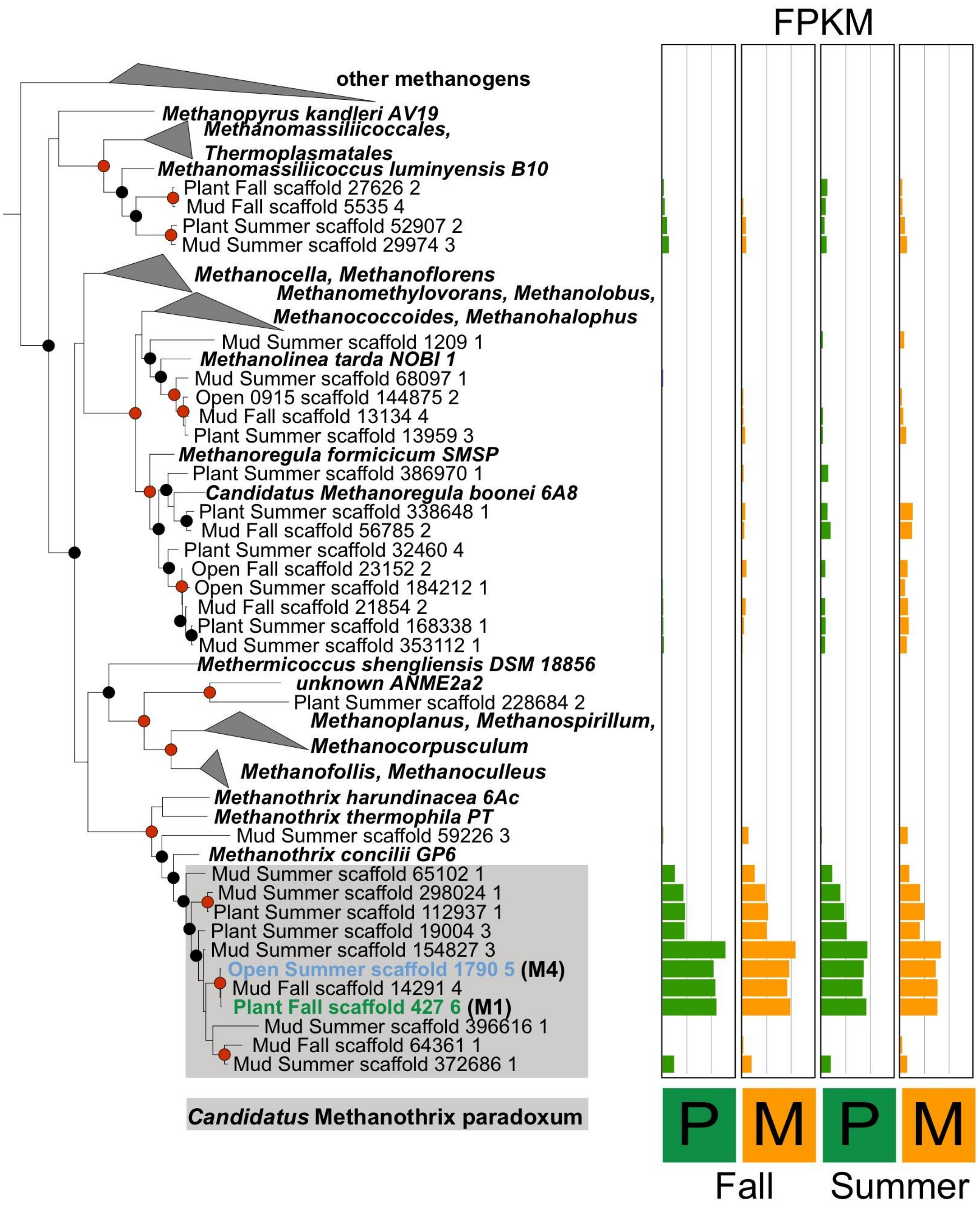
FPKM
other methanogens
Methanopyrus kandleri AV19
Methanomassiliicoccales, Thermoplasmatales
Methanomassiliicoccus luminyensis B10
Plant Fall scaffold 27626 2
Mud Fall scaffold 5535 4
Plant Summer scaffold 52907 2
Mud Summer scaffold 29974 3
Methanocella, Methanoflorens
Methanomethylovorans, Methanolobus, Methanococcoides, Methanohalophus
Mud Summer scaffold 1209 1
Methanolinea tarda NOBI 1
Mud Summer scaffold 68097 1
Open 0915 scaffold 144875 2
Mud Fall scaffold 13134 4
Plant Summer scaffold 13959 3
Methanoregula formicicum SMSP
Plant Summer scaffold 386970 1
Candidatus Methanoregula boonei 6A8
Plant Summer scaffold 338648 1
Mud Fall scaffold 56785 2
Plant Summer scaffold 32460 4
Open Fall scaffold 23152 2
Open Summer scaffold 184212 1
Mud Fall scaffold 21854 2
Plant Summer scaffold 168338 1
Mud Summer scaffold 353112 1
Methermicoccus shengliensis DSM 18856
unknown ANME2a2
Plant Summer scaffold 228684 2
Methanoplanus, Methanospirillum, Methanocorpusculum
Methanofollis, Methanoculleus
Methanothrix harundinacea 6Ac
Methanothrix thermophila PT
Mud Summer scaffold 59226 3
Methanothrix concilii GP6
Mud Summer scaffold 65102 1
Mud Summer scaffold 298024 1
Plant Summer scaffold 112937 1
Plant Summer scaffold 19004 3
Mud Summer scaffold 154827 3
Open Summer scaffold 1790 5 (M4)
Mud Fall scaffold 14291 4
Plant Fall scaffold 427 6 (M1)
Mud Summer scaffold 396616 1
Mud Fall scaffold 64361 1
Mud Summer scaffold 372686 1
Candidatus Methanothrix paradoxum
P
M
P
M
Fall
Summer

Supplementary Figure 7 | **Mapping of metatranscript reads to methanogen diversity sampled in the metagenomic dataset shows *Candidatus* Methanothrix paradoxum are responsible for a majority of *mcrA* transcripts in oxic soils.** On the left, a phylogenetic tree with *mcrA* reference nucleotide sequences from isolated methanogens (bold) and these wetland metagenome-derived sequences denoted by ecosite (black, not bold). *Candidatus* Methanothrix paradoxum *mcrA* sequences are shown in the grey box, two of which were recovered in genome bins (denoted M1, M4, colored). Metatranscripts from plant (P) and mud (M) ecosites in fall and summer were mapped to this *mcrA* sequence database. The bar chart on the right summarizes the normalized transcript abundance (scale from 0-150,000 fragments per kilobase per million mapped (FPKM)). Data are the average from triplicate cores collected in each ecosite and season. *Methanothrix* account for 84% of the recruited *mcrA* metatranscript reads. Bootstrap values ≥75 (black), or 100 (red) are denoted by circle color on the node. Collapsed node "other methanogens" contains nucleotide sequences from the genera *Methanobacterium, Methanobrevibacter, Methanocaldococcus, Methanococcus, Methanosphaera, Methanothermobacter, Methanothermococcus, Methanothermus,* and *Methanotorris.* The input FASTA file used to generate this phylogenetic analysis (Supplementary Data 9) and the mapping results (Supplementary Data 3) are provided.

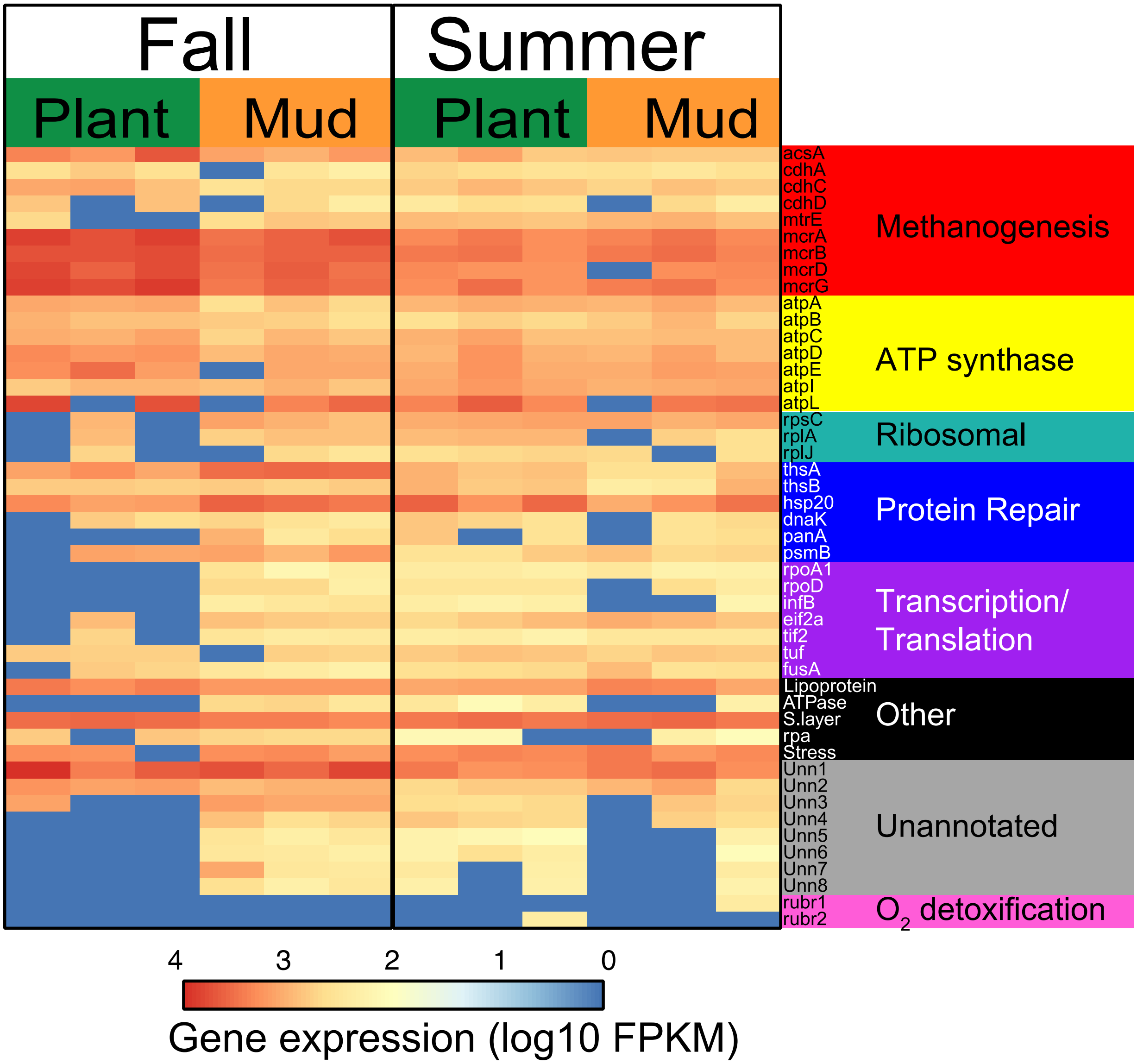

Supplementary Figure 8 | ***Candidatus* Methanothrix paradoxum (genome M1) transcript abundance patterns shared across seasons and ecosites.** Log_{10}FPKM values are shown for each replicate transcriptome for a subset of genes. Gene abbreviations are shown with assignment to functional categories performed manually (Supplementary Data 3). Beyond genes in the pathway for methanogenesis pathway (red) other genes with high transcript relative abundance include those for energy generation (yellow), protein production and repair (green and blue), cell surface (other), and unannotated genes (grey). Despite encoding multiple oxygen detoxification mechanisms (Supplementary Data 2), we show the two highest expressed oxygen detoxification genes (pink) were not comparatively highly or consistently transcribed by ecosite or season (Supplementary Note 3). The mapping results used to construct this figure are provided (Supplementary Data 3), as well as a complete list of M1 genes (Supplementary Data 5).

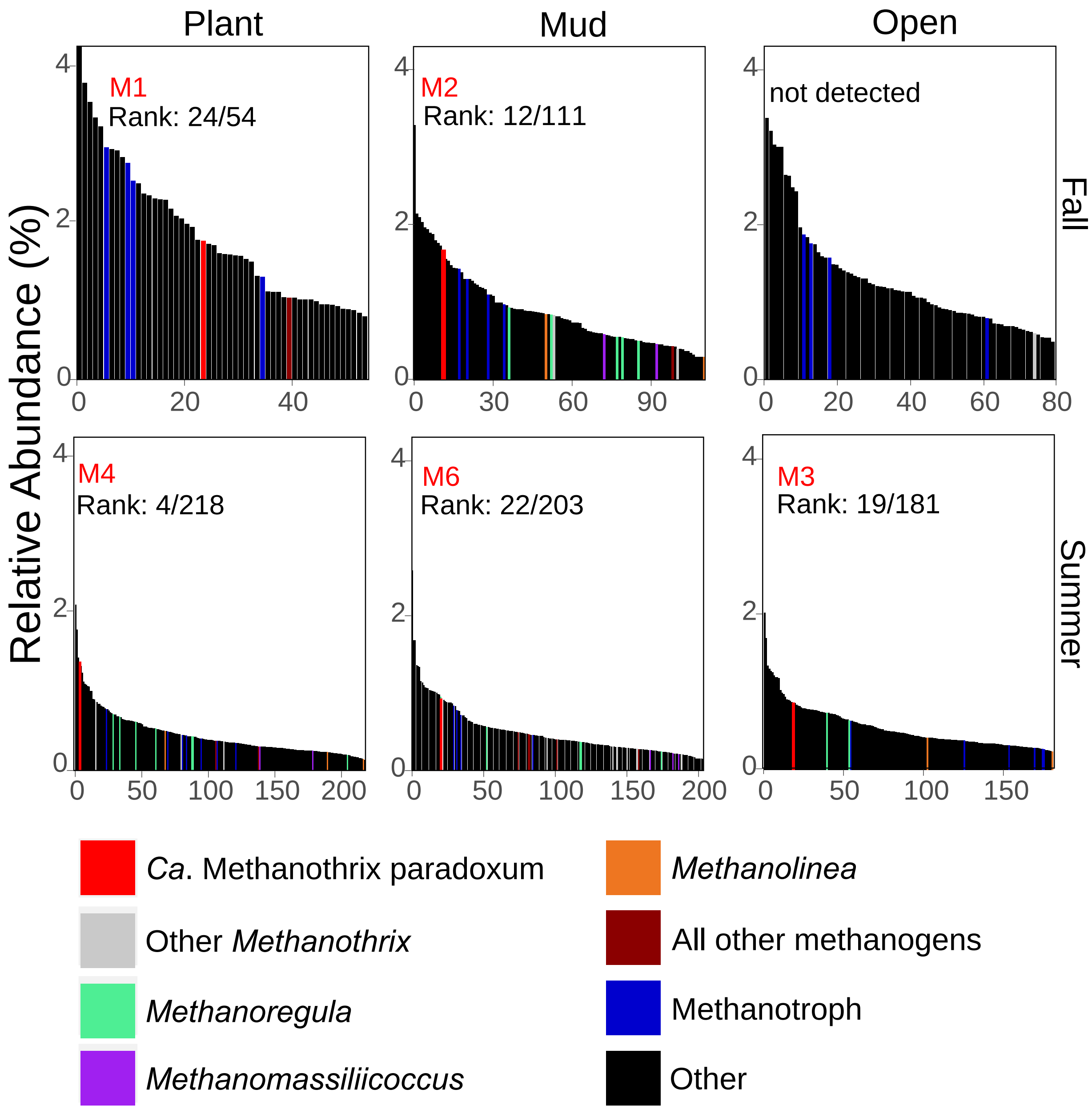

Supplementary Figure 9 | ***Candidatus* Methanothrix paradoxum are dominant methanogens and often dominant members of surface soil communities.** Rank abundance curves for the microbial community from surface (0-5 cm) soil metagenomes. The y-axis depicts the percent relative abundance of the *rpsC* (30S ribosomal protein S3) gene in the assembled metagenome. The rank and relative abundance of *Candidatus* Methanothrix paradoxum *rpsC* genes in each sample is denoted in red color and summarized in the left corner. The relative abundance of *rpsC* genes assigned to methane cycling organisms are also denoted: other *Methanothrix* (grey), *Methanoregula* (green), *Methanomassiliicoccus* (purple), *Methanolinea* (orange), all other methanogens (crimson), and methanotrophs (blue) are also shown.

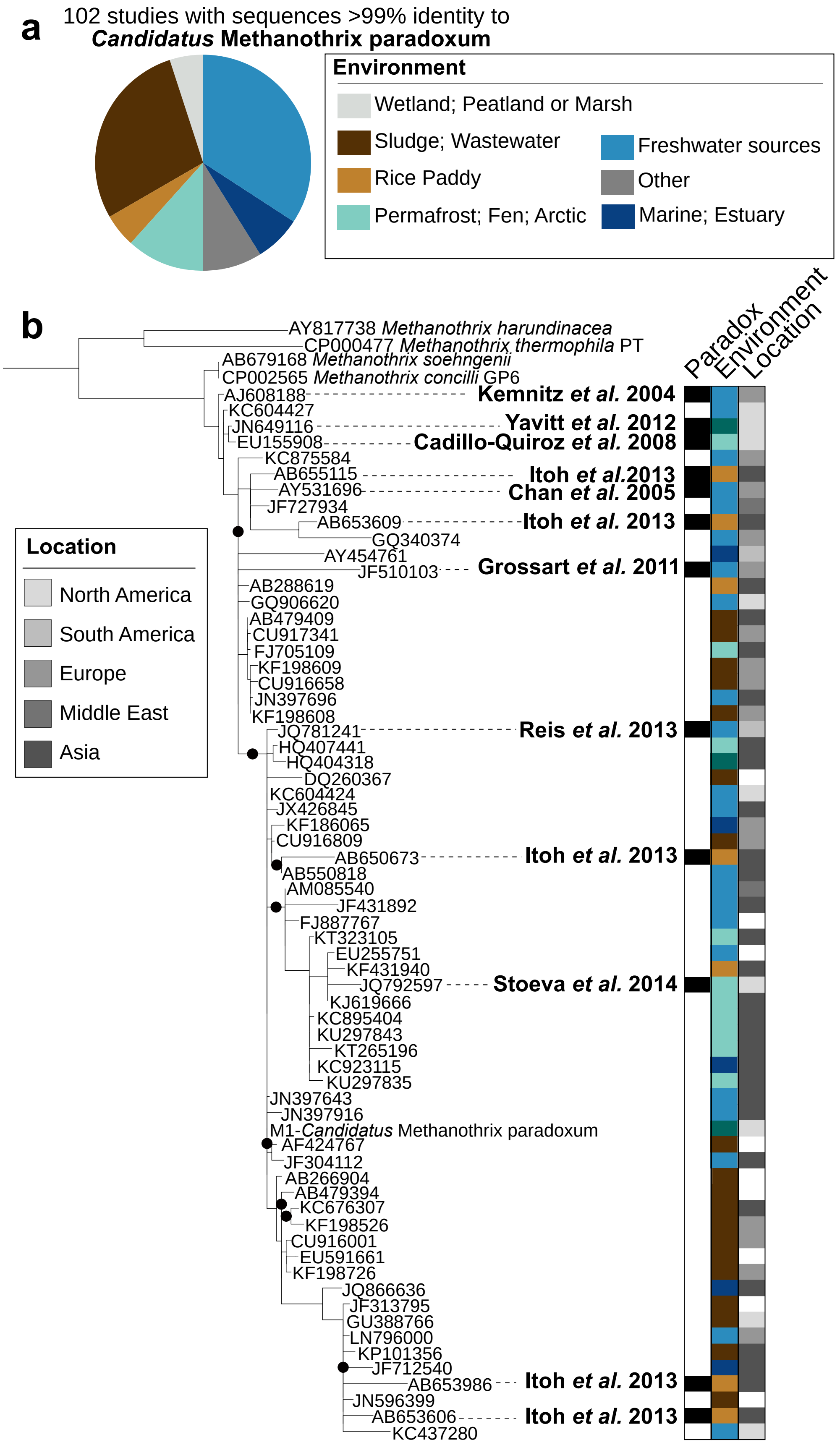

a
102 studies with sequences >99% identity to
Candidatus Methanothrix paradoxum
Environment
Wetland; Peatland or Marsh
Sludge; Wastewater
Rice Paddy
Permafrost; Fen; Arctic
Freshwater sources
Other
Marine; Estuary
b
Paradox
Environment
Location
Location
North America
South America
Europe
Middle East
Asia
AY817738 *Methanothrix harundinacea*
CP000477 *Methanothrix thermophila* PT
AB679168 *Methanothrix soehngenii*
CP002565 *Methanothrix concilli* GP6
AJ608188 Kemnitz *et al.* 2004
KC604427
JN649116 Yavitt *et al.* 2012
EU155908 Cadillo-Quiroz *et al.* 2008
KC875584
AB655115 Itoh *et al.*2013
AY531696 Chan *et al.* 2005
JF727934
AB653609 Itoh *et al.* 2013
GQ340374
AY454761
JF510103 Grossart *et al.* 2011
AB288619
GQ906620
AB479409
CU917341
FJ705109
KF198609
CU916658
JN397696
KF198608
JQ781241 Reis *et al.* 2013
HQ407441
HQ404318
DQ260367
KC604424
JX426845
KF186065
CU916809
AB650673 Itoh *et al.* 2013
AB550818
AM085540
JF431892
FJ887767
KT323105
EU255751
KF431940
JQ792597 Stoeva *et al.* 2014
KJ619666
KC895404
KU297843
KT265196
KC923115
KU297835
JN397643
JN397916
M1-*Candidatus* Methanothrix paradoxum
AF424767
JF304112
AB266904
AB479394
KC676307
KF198526
CU916001
EU591661
KF198726
JQ866636
JF313795
GU388766
LN796000
KP101356
JF712540
AB653986 Itoh *et al.* 2013
JN596399
AB653606 Itoh *et al.* 2013
KC437280

Supplementary Figure 10 | ***Candidatus* Methanothrix paradoxum is globally distributed in a variety of ecosystems.** **A.** 868 16S rRNA genes from 102 studies were recovered from public databases that were >99% similar to the *Candidatus* Methanothrix paradoxum 16S rRNA gene from genome bin M1. Pie chart represents studies where *Candidatus* Methanothrix paradoxum was detected, shown by environment type (Supplementary Data 4, Supplementary Note 4). **B.** Maximum likelihood phylogenetic tree constructed with representative 16S rRNA sequences identified in A, with black node circles indicating bootstrap values >70%. The 'environment' where the sequence was recovered from is denoted by color. Geographic 'location' is indicated in greyscale. Importantly, several *Methanothrix* sequences detected under oxic conditions or where the methane paradox was cited are noted in black under 'paradox'. References for these papers are provided by first authors last name and year of publication[5-12]. Taken together with our other data (e.g. Supplementary Figs 5,6) the new species of *Methanothrix* we propose here is globally distributed and active in high methane-flux environments, suggesting this lineage may be a predominant contributor to global methane production in anoxic and oxic environments. The input FASTA file used to generate this figure (Supplementary Data 10) and the metadata (Supplementary Data 4) are provided.

Supplementary Table 1 | Metagenomic and metatranscriptomic sample sequencing data

Data type - Same name	Read Length (bp)	Read count	Total Sequencing (Gbp)
Metagenome - Fall Plant	100	98581091	19.72
Metagenome - Fall Mud	100	208578991	41.72
Metagenome - Fall Open	100	96172864	19.23
Metagenome - Summer Plant	151	276581518	83.53
Metagenome - Summer Mud	151	231981210	70.06
Metagenome - Summer Open	151	231999210	70.06
Metatranscriptome - Fall Plant 1	151	121148486	36.59
Metatranscriptome - Fall Plant 2	151	101678848	30.71
Metatranscriptome - Fall Plant 3	151	135941546	41.05
Metatranscriptome - Fall Mud 1	151	138053478	41.69
Metatranscriptome - Fall Mud 2	151	128188682	38.71
Metatranscriptome - Fall Mud 3	151	122537220	37.01
Metatranscriptome - Summer Plant 1	151	115335708	34.83
Metatranscriptome - Summer Plant 2	151	138225486	41.74
Metatranscriptome - Summer Plant 3	151	129084508	38.98
Metatranscriptome - Summer Mud 1	151	134446440	40.60
Metatranscriptome - Summer Mud 2	151	118650014	35.83
Metatranscriptome - Summer Mud 3	151	143765256	43.42

Supplementary Table 2 | *Methanothrix* genome bin characteristics

Genome name (Short)	Genome Name (Full)	Season	Land Coverage Type	Completion	Single copy gene overages	Largest contig	Assembled length in bin	Number of contigs in bin
M1	M1-NSP1	Fall	Plant	90%	2.9%	23,510	1,471,312	252
M2	M2-NSM2	Fall	Mud	91%	1.9%	38,789	1,751,596	238
M3	M3-ASO1	Summer	Water	76%	4.8%	14,739	1,170,295	263
M4	M4-ASP1-1	Summer	Plant	57%	3.8%	10,710	688,846	179
M5	M5-ASP1-2	Summer	Plant	31%	5.7%	5,525	208,261	68
M6	M6-ASM2	Summer	Mud	79%	3.8%	21,054	1,171,139	249

Supplementary Note 1 - Greenhouse gas emissions and estimates

The concentration profile of methane is not directly indicative of the vertical distribution of microbial activity in the soil because changes in methane concentration profiles between any two points time are due to several processes: 1) microbial activity (i.e., methanogenesis and methanotrophy), 2) transport between soil layers, and 3) flux between the soil layers and the atmosphere. To isolate the effect of microbial activity, it is therefore necessary to account for the transport of methane, both between soil layers and leaving the system. Transport within layers can be caused by molecular diffusion, bulk flow of porewater, ebullition, and via plants. In permanently flooded soils, bulk flow can be assumed to be negligible, reducing the problem in complexity. Transport out of the soil has been documented to occur in one of three ways: 1) molecular diffusion, 2) plant transport, and 3) ebullition[13].

Chamber measurements of fluxes were used as the upper boundary condition for the diffusion model whose results are show in Figure 1 of the main text. Chamber flux measurements are filtered for ebullition and so are representative only of diffusion as an egress mechanism. By focusing on mud and open water, we were thus able to disregard plant transport. We disregarded ebullition both between soil layers and out of the soil column from this analysis. This makes our estimates a lower boundary on how much methane could be produced in oxic soils. This is because ebullition is effectively a numerically unaccounted for sink term in the upper layers. If this sink term were to be quantified and accounted for in the analysis, the microbial activity term (*R*) in the affected layers would necessarily have to be increased to compensate for the methane lost. CH_4 moved from lower layers as bubbles also may not be directly emitted as gases in bubbles are often reabsorbed as dissolved gases during transport, limiting the impact of this simplifying assumption.

Supplementary Note 2 - Metagenomic and metatranscriptomic analyses

Metagenomic assembly and binning of these surface soil samples yielded 58 bins, with eight identified as methanogens, and six of these as *Candidatus* Methanothrix paradoxum. The other sampled methanogen bins (48% and 61% complete) were most closely related to *Methanoregula* spp., which were less abundant and less active methanogens of the surface soil community sampled here. *Candidatus* Methanothrix paradoxum genomic bin quality and completion are summarized below, but ranged from 31-91% estimated completeness, with low numbers of overages (<3% in the most complete genomes) (Supplementary Fig. 3). The most metabolically complete genome, M1, was used as a population representative. We recovered one 16S rRNA gene fragment (1472 bp) in the most metabolically complete genome M1.

Metabolic profiling of the *Candidatus* Methanothrix paradoxum genomes was performed manually, and, to account for any misbinning, we confirmed that any gene included in the metabolic summary was supported by other genes on the contig annotated as *Methanothrix* and having similar GC and coverage to the overall bin. A summary of the metabolic capabilities and the gene transcripts detected is included (Supplementary Data 2). All of the pathways required for acetoclastic methanogenesis were present and highly transcribed across both ecosites and seasons, a finding consistent with our pore-water substrate data that showed a significant positive correlation between acetate concentrations and *mcrA* transcripts (Supplementary Fig. 2B). On the other hand, essential genes for methanol and methylamine activation or utilization were not present, while genes for hydrogen utilization were present but not transcribed. These findings reflect a lack of significant correlation between *mcrA* transcripts and these other methanogenic

substrates in our porewater (Supplementary Fig. 2B). Based on this data, and other reports from previously characterized *Methanothrix* spp.[14,15], we consider it likely that *Candidatus* Methanothrix paradoxum is also an obligate acetoclastic methanogen.

To identify *Candidatus* Methanothrix paradoxum genes that were highly expressed across both seasons, we identified the top 100 transcribed genes in each season, resulting in 140 unique genes from summer and fall. To show gene transcription patterns shared across both seasons, the log10 FPKM for each season was plotted, and 73% (102) of these genes were found to share high levels of transcription in both summer and fall surface soils (Figure 3B, white oval). These findings clearly show that key genes in the methanogenesis pathway are highly transcribed in surface soils. Additional indicators of *Candidatus* Methanothrix paradoxum activity in these oxic surface soils include the high relative abundance of transcripts for protein synthesis (transcription and translation) and energy generation (ATP synthesis). Notably, genes for protein repair (e.g. chaperones) were also highly transcribed, suggesting protein turnover and repair may be a mechanism of oxidative protection of proteins used by methanogens in oxic soils.

To gain insight into the environmental distribution of *Candidatus* Methanothrix paradoxum genomes, both the phylogenetic marker gene 30S small subunit ribosomal protein 3 *(rps3)* and the functional marker gene *mcrA* found within the genome M1 were used to query environmental metagenomes available on the Joint Genome Institute Integrated Microbial Genomes/Microbiome (JGI-IMG/M December 2016). These analyses clearly show that genotypes similar to the reconstructed genomes here are present in other hydric soils from Barrow, Alaska and Twitchell Island, California. Also of interest, similar to our reports that *Candidatus* Methanothrix paradoxum *mcrA* and *rpsC* genes were highly transcribed in both seasons and ecosites (Fig. 3C), these genes were also highly transcribed in surface soils from Twitchell Island, clearly demonstrating that *Candidatus* Methanothrix paradoxum can contribute to methane cycling across geographically distinct wetlands (Supplementary Figs 5 and 6).

Supplementary Note 3 - Comparative *Methanothrix* genomic analyses

Here we expand on the main text and provide an inventory of the oxygen tolerance genes reported in other methanogens and mined from *Methanothrix* genomes: thioredoxin / thioredoxin reductase (t*rx* and *trxr*), rubrerythrin (*rbr*), peroxiredoxin (*prx*), desulfoferrodoxin (*dfx*), rubredoxin (*rbx*), glutaredoxin (*grx*), peroxidase (*px*), catalase (*kat*), superoxide dismutase (*sod*), $F_{420}H_2$ oxidase (*fprA*), and cytochrome D oxidase (*cyd*)[16]. From our wetland genomes we recovered known oxygen detoxification genes, including those for stabilizing free radicals (*sod*), reducing toxic reactive oxygen species (*kat, px, rbr, rbx*), and for repairing oxidative disulfide damage (*prx* and *trx*). These data are summarized in Supplementary Data 2. Also, in contrast to prior reports of methanogens in oxic laboratory experiments, no oxygen tolerance genes were expressed abundantly (single sample >1000 FPKM) or consistently (present >3 samples) in our field data.

It is possible that *Methanothrix* confinement to anoxic microenvironments with the bulk oxic layer may be a possible explanation for the activity of methanogens in our wetland surface soils. One anoxic microhabitat would be the formation of biofilms. While numerous publications have reported on the presence and contributions of *Methanothrix* in biofilms from diverse systems, including pipes[17], wastewater digestors[18,19], and even carbonate chimneys[20], very little work has examined the specific genes involved in biofilm formation. A collection of genes implicated in methanogen biofilm formation or conditions[21-23], as well as more general bacterial biofilm genes[24-26], were queried to the two most complete genomes of *Candidatus* Methanothrix

paradoxum and to the community metatranscriptome. None of these biofilm-associated genes (1e-5 identity) were recovered in *Candidatus* Methanothrix paradoxum genomes, nor were these biofilm genes detected transcribed in the community metatranscriptomes.

Of course, we cannot rule out contributions from yet-annotated and highly expressed genes in *Candidatus* Methanothrix paradoxum in oxygen adaptation, especially since some of these were potential s-layer or extracellular proteins. Lastly, metatranscript recruitment plots between our most complete genome and the nearest *Methanothrix* neighbor (*M. concilii*) demonstrated recruitment of a log-fold more genes using our wetland genotypes. This finding showcases the value of reconstructed genomes when functionally profiling *in situ* metabolisms from soils or other habitats containing high numbers of uncultivated or genomically undersampled strains.

Supplementary Note 4 - *Candidatus* Methanothrix paradoxum biogeography

Based on the data collected here and our prior study[1], we sampled the microbial community in these wetland soils by 16S rRNA sequencing for two years (Nov 2013, Nov 2014, Feb 2015, March 2015, Aug 2015) and from multiple ecosites (n=126). These samples spanned a range of depths, with 23.8% being collected from the first 12 cm surface soils. Across these samples we recovered 1560 (Nov 2013) and 5663 (remaining dates) bacterial and archaeal OTUs (defined at 97% nucleotide identity). Seven of these OTUs were classified as belonging to the genus *Methanothrix*, but due to short amplicon size further taxonomic resolution was not possible (V4 16S rRNA region, ~300 bp).

The 16S rRNA data from mud and open water samples (n=60), published previously, used a larger amplicon size (V3-V6 region of the 16S rRNA gene) and targeted the Archaeal 16S rRNA with archaeal-specific primers, allowing for greater phylogenetic resolution and deeper sampling of *Methanothrix* strains[1]. Consistent with our metagenomic findings from surface soils (Supplementary Fig 9), OTUs representing *Methanothrix* spp. (max relative abundance 47%, mean 21% +/- 8%) and hydrogenotrophic *Methanoregula* spp. (max relative abundance 10%, mean 4% +/- 2%) were the two most abundant methanogens across the wetland[1]. Additionally, we observed OTU-level differences in abundance along soil depth gradients. One *Methanothrix* OTU (OWC_a1), which was 100% identical to the 16S rRNA gene recovered from our *Candidatus* Methanothrix paradoxum genome, was enriched in the surface soils relative to the deep (mean 7% +/-6% greater within-core relative abundance in shallow samples vs. deep samples) and represented the most abundant archaea in surface soils[1]. These 16S rRNA gene results from a prior year, and from more ecosites, support our metagenomic rank abundance curves (Supplementary Fig 9) and suggests findings generated here may extend much more broadly across larger spatial and temporal time scales in these wetland soils.

The surface-enriched *Candidatus* Methanothrix paradoxum 16S rRNA gene from the M1 genome has 99% or greater nucleotide similarity to sequences found globally in 102 studies representing a variety of ecosystems (Supplementary Fig. 10). The distribution of these studies includes 28% wastewater, 34% freshwater (dominated by lake waters), 7% estuary or marine, 8% permafrost (with equal distribution across mountain and arctic/boreal), and 10% wetlands (including rice paddy, peatland, marsh) (Supplementary Data 4). We acknowledge that this distribution is largely affected by the sampling of these ecosystems, but the data highlight the broad relevance of *Candidatus* Methanothrix paradoxum across ecosystems and geographic regions. A subset of representative sequences from this survey is included in a phylogenetic analysis (Supplementary 9, Supplementary Data 4).

From this meta-analysis, notable was the prevalence *Candidatus* Methanothrix paradoxum in surface soils (often oxygenated), including tropical streams[11], arctic wetlands[12], and temperate peatlands[7,8]. Moreover, we show representatives similar to *Candidatus* Methanothrix paradoxum were present in prior soil and lake studies where the methane paradox was suggested (Supplementary Figure 10). Of particular interest, particle-associated *Methanothrix* (some of which were highly similar to *Candidatus* Methanothrix paradoxum) were inferred to be responsible for methane production in oxic lake waters, one of the first methane paradox publications[5]. In terrestrial systems, *Candidatus* Methanothrix paradoxum were also enriched and inferred to be active in the top 5 cm of soils[12,27-30], some of which were shown to be oxic and have high numbers of transcripts from *mcrA* belonging to *Methanothrix*[27]. Collectively these findings, in light of our results, suggest *Candidatus* Methanothrix paradoxum may be a critical driver of methanogenesis in oxic habitats from both aquatic and terrestrial systems.

Supplementary Note 5

Here we based our site level scaling on a method similarly used by Bogard *et al.*[30], which determined the contribution of oxic methanogenesis to overall lake methane flux. We estimate that between 40 and 90% of methane emissions across the site is driven by oxic soil production. Quantification of the proportion of emitted methane due to generation in the oxic soil zone is a non-trivial problem and we acknowledge that there are some coarse assumptions made in our estimate, which we discuss below. The rates of methane emission observed from the 3 ecosites were at the high end of the rates reported in similar wetlands[31].

The net methane activity values generated in this study (Fig. 1) result from methanogenesis and methanotrophy at each soil layer. The individual contributions of these processes to our predicted net activity values are unknown and to partition one must make assumptions on how oxygen and alternate electron acceptors affect these rates. Net negative activity layers in the soil almost certainly still have methanogenesis, but the rate is lower than co-located methanotrophy. However, some of the generated methane may be mobilized towards the soil/water interface before the full amount is consumed (displaced by incoming methane from other layers). In order to be emitted, methane generated in the deep layers must pass through the oxic zone, which may well decrease its effective transmission to the atmosphere as large portions of it may be consumed in methanotrophic reactions as it passes through.

Furthermore, positive net methane activity values in the shallow layers are here treated purely as methanogenesis. In reality, there must be methanotrophy in these layers but methanogenesis must be high enough to offset this sink in order to produce the activity levels we observe. Future quantification should include detailed modeling of observed tracers or in depth isotopic analysis[32] to provide more comprehensive accounting of the origin of emitted CH_4.

Supplementary Discussion

Several mechanisms have been proposed to explain methane production in oxic habitats, here we discuss these hypothesis in light of our data. First, based on the increased methanogenesis activity, organismal abundance, and methane production in oxic soils, we conclude that diffusion from methanogen activity in deeper anoxic layers[33,34] is not a major contributor in our system. Similarly, our biological and modeling evidence does not support methane produced from UV-irradiated plants[35] or as a by-product of heterotrophic decomposition[36,37]. As additional evidence that this process is driven by methanogens and not via microbial decomposition of methylated compounds[36], we failed to detect methylphosphonate and

its derivatives in our NMR porewater metabolite data, and we failed to detect *phnJ* transcripts (or any *phn* subunits involved in this pathway) in our community metatranscriptomics data[37-39].

Moreover, while it has been suggested in other ecosystems that methanogens may find oxygen shelter inside protozoans, we failed to detect 18S rRNA sequences from any known methanogen ciliate hosts in our EMIRGE reconstructions[40,41], nor did we find consistent eukaryotic signal correlating to methanogen activity in our metagenomic data. This signifies that it is unlikely that endosymbiont methanogens are the primary source of methane in these soils. Here we show methanogenesis activity in oxic soils is driven by canonical, likely free-living methanogens.

Our data is the first methane paradox study to show which methanogens are transcriptionally active in bulk oxic habitats. Based on our metatranscript data that demonstrates i) multiple methanogens can be active in oxic soils and ii) that failed to identify a known genetic mechanism explaining increased activity of *Methanothrix* (e.g. oxygen tolerance, oxygen detoxification), we consider it plausible that surface soil methanogens may not be encountering the high levels of oxygen measured in porewaters.

Quantification of anoxic microsites in soils and the mechanisms sustaining these zones represents areas for future research. When considering explanations for the increased activity of methanogens in bulk oxygenated soils, it could be possible that analogously to wastewater digesters and microbial fuel cells, active carbon decomposition by other members of the community produce local anoxic conditions favorable for methanogens[42,43]. Analogously, Bogard *et al* suggested that fermentative bacteria create the conditions for anoxic methanogenesis in oxic surface lake waters[30]. In wetland soils, it is also recognized that the combination of labile dissolved organic matter (DOM) and reduced rates of gas diffusion through saturated soil pores can facilitate the formation of anoxic microsites that fuel other anaerobic metabolisms (e.g. nitrate and iron reduction) in bulk oxic soils[44-46].

Along these lines, we consider it likely that increased lability and input of dissolved DOM from overlaying plants or surface waters contributed to the increased methanogenic activity in surface (0-5 cm) relative to deeper (>20 cm) soils. In support of this possibility, prior reports of DOM from porewaters in these wetland soils demonstrated structural differentiation with depth, with DOM molecular weight and aromaticity increasing with a depth below 5 cm[47]. In surface soils, this possible increased carbon input and decomposition (due to increased electron accepting capacity of these soils) may have contributed to the significantly greater (~on average twice as much) acetate we detected in surface compared to deep soils. We note that acetate is a non-conservative substrate, with a presumed high rate of turnover in oxygenated soils, thus the absolute concentrations in the soil may be an underestimation of methanogen substrate availability. Thus, DOM input and composition in surface soils could fuel local regions of heterotrophy, leading to oxygen consumption and the generation of acetoclastic methanogen substrates, together facilitating methane production in surface soils.

Increased concentrations of methanol and formate detected in deeper soils (Supplementary Data 1, worksheet 1) indicates methanogenesis in these soils is not likely substrate limitation. However, we and others[47] have shown that deeper soils have increased Fe(II) concentrations [Supplementary Data 1, worksheet 1], which could directly or indirectly impact methanogen activity. Directly, using pure cultures and soil measurements, methanogenesis was shown to be inhibited by addition of amorphous $Fe(OH)_3$ and humic acids[48,49]. Indirectly, increased metal ion chelation or absorption to the soil matrix in deeper soils could limit availability of required methanogen co-factors (e.g F_{430})[50]. Biological competition for

substrates[51,52], vitamins, or cofactors[53] with other microbial taxa or viral predation[54,55] could further constrain methanogens in deep soils. Ongoing research coupling integrated biogeochemical, molecular DOM characterization, and omics technologies is required to better understand the factors facilitating methanogenesis in these soil horizons.

Regardless of the mechanism, our findings that methanogenesis occurs in oxygenated soils and contributes significantly to wetland wide methane flux has important ramifications for modeling efforts. Models that simulate methane production assume down-regulation of methanogenesis in these soil layers due to oxygen concentrations, underestimating methane emissions. As a consequence, soil conditions are diagnosed as appropriate for methane production at greater depths and after longer flooding periods than are actually observed. Further understanding of microsite evolution may alter the perceived sensitivity of methane emissions to air and water temperatures, as shallower sites show higher temperature fluctuations that correlate more strongly with air temperature than deeper soil layers. It may therefore be critical to account for these processes in biogeochemical models to improve predictability of net wetland methane emissions and their effects on climate.

Supplementary References

1. Narrowe, A. B. *et al*. High-resolution sequencing reveals unexplored archaeal diversity in methane-emitting freshwater wetland soils. *Environmental Microbiology* **19**, 2192-2209 (2017)
2. Yuan, Y., Conrad, R. & Lu, Y. Transcriptional response of methanogen mcrA genes to oxygen exposure of rice field soil. *Environmental Microbiology Reports* **3**(3), 320-328 (2011)
3. Rey-Sanchez, A. C., Morin, T. H., Stefanik, K. C., Wrighton, K. C., & Bohrer, G. Determining total emissions and environmental drivers of methane flux in a Lake Erie estuarine marsh. *Ecological Engineering* (in press) (2017)
4. He, S. *et al.* Patterns in wetland microbial community composition and functional gene repertoire associated with methane emissions. *mBio* **6**(3), e00066-15 (2015)
5. Grossart, H. P., Frindte, K., Dziallas, C., Eckert, W. &Tang, K. W. Microbial methane production in oxygenated water column of an oligotrophic lake. *Proceedings of the National Academy of Sciences* **108**(49), 19657-19661 (2011)
6. Kemnitz, D., Chin, K-J., Bodelier, P., & Conrad, R. Community analysis of methanogenic archaea within a riparian flooding gradient. *Environmental Microbiology* **6**(5), 449-461 (2004)
7. Yavitt, J. B., Yashiro, E., Cadillo-Quiroz, H. & Zinder, S. H. Methanogen diversity and community composition in peatlands of the central to northern Appalachian Mountain region, North America. *Biogeochemistry* **109**, 117–131 (2012)
8. Cadillo-Quiroz, H., Yashiro, E., Yavitt, J. B. & Zinder, S. H. Characterization of the archaeal community in a minerotrophic fen and terminal restriction fragment length polymorphism-directed isolation of a novel hydrogenotrophic methanogen. *Applied Environmental Microbiology* **74**, 2059–2068 (2008)
9. Itoh, H. *et al*. Seasonal transition of active bacterial and archaeal communities in relation to water management in paddy soils. *Microbes and Environments* **28**(3), 370-380 (2013)
10. Chan, O. C. *et al*. Vertical distribution of structure and function of the methanogenic archael community in Lake Dagow sediment. *Environmental Microbiology* **7**(8), 1139-1149 (2005)

11. Reis, M. P., Barbosa, F. A. R., Chartone-Souza, E., & Nascimento, A. M. A. The prokaryotic community of a historically mining-impacted tropical stream sediment is as diverse as that from a pristine stream sediment. *Extremophiles* **17**, 301–309 (2013)
12. Stoeva, M.K. *et al*. Microbial community structure in lake and wetland sediments from a high arctic polar desert revealed by targeted transcriptomics. *PLoS One* **9**, e89531 (2014)
13. Riley, W. J. *et al*. Barriers to predicting changes in global terrestrial methane fluxes: analyses using CLM4Me, a methane biogeochemistry model integrated in CESM. *Biogeosciences* **8**(7), 1925-1953 (2011)
14. Patel, G. B. Characterization and nutritional properties of *Methanothrix concilii* sp. nov. , a mesophilic, aceticlastic methanogen. *Canadian Journal of Microbiology* **30**, 1383-1396 (1984)
15. Ma, K., Liu, X., & Dong, X. *Methanosaeta harundinacea* sp. nov., a novel acetate-scavenging methanogen isolated from a UASB reactor. *International Journal of Systematic and Evolutionary Microbiology* **56**(1), 127-131 (2006)
16. Jasso-Chávez, R. *et al*. Air-adapted *Methanosarcina acetivorans* shows high methane production and develops resistance against oxygen stress. *PloS one* **10**(2), e0117331 (2015)
17. Gomez-Alvarez, V., Revetta, R. P., & Domingo, J. W. S. Metagenome analyses of corroded concrete wastewater pipe biofilms reveal a complex microbial system. *BMC Microbiology* **12**, 122 (2012)
18. Fernández, N., Díaz, E. E., Amils, R., & Sanz, J. L. Analysis of microbial community during biofilm development in an anaerobic wastewater treatment reactor. *Microbial Ecology* **56**, 121-132 (2008)
19. McKeown, R. M. et al. Psychrophilic methanogenic community development during long-term cultivation of anaerobic granular biofilms. *International Journal of Systematic and Evolutionary Microbiology* **3**, 1231-1242 (2009)
20. Brazelton, W. J., Mehta, M. P., Kelley, D. S., & Baross, J. A. Physiological differences within a single-species biofilm fueld by serpentinization. mBio **2**(4), e00127-11 (2011)
21. Zhou, L. *et al*. Transcriptomic and physiological insights into the robustness of long filamentous cells of *Methanosaeta harundinacea*, prevalent in upflow anaerobic slude blanket granules. *Applied and Environmental Microbiology* **81**, 831-839 (2015)
22. Mandal, A. K., Cheung, W. D., and Argüello, J. M. Characterization of a thermophilic p-type Ag+/Cu+-ATPase from the extremophile *Archaeoglobus fulgidus*. *Journal of Biological Chemistry* **277**(9), 7201-7208 (2002)
23. Zhang, G. *et al*. Acyl homoserine lactone-based quorum sensing in a methanogenic archaeon. *International Journal of Systematic and Evolutionary Microbiology* **6**, 1336-1344 (2012)
24. Flemming, H.-C. & Wingender, J. The biofilm matrix. *Nature Review Microbiology* **8**(9), 623-633 (2010)
25. Ma, L. et al. Assembly and development of the *Pseudomonas aeruginosa* bioflilm matrix. *PLoS Pathogens* **5**(3), e10000354 (2009)
26. Watnick, P. I. & Kolter, R. Steps in the development of a *Vibrio cholera* El Tor biofilm. *Molecular Microbiology* **34**(3), 586-595 (1999)
27. Franchini, A. G., Henneberger, R., Aeppli, M. & Zeyer, J. Methane dynamics in an alpine fen: a field-based study on methanogenic and methanotrophic microbial communities. *FEMS Microbiology Ecology* **91**(3), fiu032 (2015)

28. Liu, D. *et al*. Effect of paddy-upland rotation on methanogenic archaeal community structure in paddy field soil. *Microbial ecology* **69**(1), 160-168 (2015)
29. Lee, C. G., Watanabe, T., Murase, J., Asakawa, S. & Kimura, M. Growth of methanogens in an oxic soil microcosm: Elucidation by a DNA-SIP experiment using 13 C-labeled dried rice callus. *Applied Soil Ecology* **58**, 37-44 (2012)
30. Bogard, M. J. *et al*. Oxic water column methanogenesis as a major component of aquatic CH4 fluxes. *Nature communications* **5**, (2014)
31. Rey-Sanchez, A. C., Morin, T. H., Stefanik, K. C., Wrighton, K. C., & Bohrer, G. Determining total emissions and environmental drivers of methane flux in a Lake Erie estuarine marsh. *Ecological Engineering* (in press) (2017)
32. Duenas, C., Fernandez, M. C., Carretero, J., Perez, M., & Liger, E. Consumption of methane by soils**.** *Environmental Monitoring and Assessment* **31**, 125–130 (1994)
33. Rudd, J. W. & Hamilton, R. D. Methane cycling in a eutrophic shield lake and its effects on whole lake metabolism. *Limnology and Oceanography* **23**(2), 337-348 (1978)
34. Bastviken, D., Cole, J., Pace, M. & Tranvik, L. Methane emissions from lakes: Dependence of lake characteristics, two regional assessments, and a global estimate. *Global Biogeochemical Cycles* **18**(4), GB4009 (2004)
35. Vigano, I., Van Weelden, H., Holzinger, R., Keppler, F. & Rockmann, T. Effect of UV radiation and temperature on the emission of methane from plant biomass and structural components, *Biogeosciences* **5**(1), 243–270 (2008)
36. Karl, D. M. *et al*. Aerobic production of methane in the sea. *Nature Geoscience* **1**(7), 473-478. (2008)
37. Repeta, D. J. *et al*. Marine methane paradox explained by bacterial degradation of dissolved organic matter. *Nature Geoscience 9*(12), 884-887 (2016)
38. Yao, M., Henny, C. & Maresca, J. A. Freshwater bacteria release methane as a by-product of phosphorus acquisition. *Applied and Environmental Microbiology* **82**(23), 6994-7003 (2016)
39. Kamat, S. S., Williams, H. J., Dangott, L. J., Chakrabarti, M. & Raushel, F. M. The catalytic mechanism for aerobic formation of methane by bacteria. *Nature* **497**(7447), 132-136 (2013)
40. Narayanan, N., Krishnakumar, B., Anupama, V. N. & Manilal, V. B. *Methanosaeta* sp., the major archaeal endosymbiont of *Metopus es*. *Research in Microbiology* **160**(8), 600-607 (2009)
41. Yarlett, N. & Hackstein, J. H. Hydrogenosomes: one organelle, multiple origins. *Bioscience* **55**(8), 657-668. (2005)
42. Scott, R. L., Williams, T. N., Whitmore, T. N., & Lloyd, D. Direct measurement of methanogenesis in anaerobic digestors by membrane inlet mass spectrometry. *European Journal of Applied Microbiology and Biotechnology* **18**, 236-241 (1983)
43. Chae, K-J *et al*. Methanogenesis control by employing various environmental stress conditions in two-chambered microbial fuel cells. *Bioresource Technology* **101**, 5350-5357 (2010)
44. Keiluweit, M., Nico, P. S., Kleber, M. & Fendorf, S. Are oxygen limitations under recognized regulators of organic carbon turnover in upland soils? *Biogeochemistry* **127**(2-3), 157-171 (2016)

45. Arah, J. R. M. & Vinten, A. J. A. Simplified models of anoxia and denitrification in aggregated and simple-structured soils. *European Journal of Soil Science* **46**(4), 507-517 (1995)
46. Sobolev, D. & Roden, E. E. Evidence for rapid microscale bacterial redox cycling of iron in circumneutral environments. *Antonie Van Leeuwenhoek* **81**(1), 587-597 (2002)
47. Chin, Y. P., Traina, S. J., Swank, C. R., & Backhus, D. Abundance and properties of dissolved organic matter in pore waters of a freshwater wetland. *Limnology and Oceanography* **43**(6), 1287-1296 (1998)
48. vanBodegom, P. M. & Stams, A. J. M. Effects of alternative electron acceptors and temperature on methanogenesis in rice paddy soils. *Chemosphere* **2**: 167-182
49. Miller, K. E., Lai, C. T., Friedman, E. S., Angenent, L. T., & Lipson, D. A. Methane suppression by iron and humic acids in soils of the arctic coastal plain. *Soil Biology and Biochemistry* **83**(17), 6e183 (2015)
50. Pramanik, P. & Kim, P. J. Effect of limited nickel availability on methane emission from EDTA treated soils: coenzyme M an alternative biomarker for methanogens. *Chemosphere* **90**(2), 873-876 (2013)
51. Roden, E. E. & Wetzel, R. G. Competition between Fe(III)-reducing and methanogenic bacteria for acetate in iron-rich freshwater sediments. *Microbial Ecology* **45**, 252-258 (2003)
52. Koysyurbenko, O. R., Glagolev, M. V., Nozhevnikova, A. N., & Conrad, R. Competition between homoacetogenic bacteria and methanogenic archaea for hydrogen at low temperature. *FEMS Microbial Ecology* **38**, 153-159 (2001)
53. Seth, E. C. & Taga, M. E. Nutrient cross-feeding in the microbial world. *Frontiers in Microbiology* **5**, 350 (2014)
54. Lai, M-C. & Chen, S-C. *Methanofollis aquaemaris* sp. nov., a methanogen isolated from an aquaculture fish pond. *International Jounal of Systematic and Evolutionary Microbiology* **51**, 1873-1880 (2001)
55. Grissa, I., Vergnaud, G., & Pourcel, C. The CRISPRdb database and tools to display CRISPRs and to generate dictionaries of spacers and repeats. *BioMed Central Bioinformatics* **8**, 172 (2007)
