# Supplementary material for: Methanogenesis in oxygenated soils is a substantial fraction of wetland methane emissions
Source: Nat Commun. 2017 Nov 16;8:1567. doi: 10.1038/s41467-017-01753-4 (PMC5691036; doi:10.1038/s41467-017-01753-4)
Supplement: Supplementary file 3 — Description of Additional Supplementary Files [file 41467_2017_1753_MOESM3_ESM.pdf]

Description of Additional Supplementary Files

File Name: Supplementary Data 1

Description: Meta-data from surface and deep soils collected in November 2014 and August 2015 from plant, open, and mud ecosites. Geochemical (worksheet 1), quantitative reverse transcriptase PCR of mRNA (worksheet 2), and August oxygen concentrations with depth (worksheet 3).

File Name: Supplementary Data 2

Description: Metabolic summary of Candidatus Methanothrix paradoxum representative genome M1.
Genes assigned to different functional categories with mapped detected transcripts (worksheet 1), a summary of methanogen oxygen tolerance genes detected in Candidatus Methanothrix paradoxum genomes (worksheet 2), a summary of the M1 genome bin (worksheet 3), and a summary of biofilm indicator genes used for analyses (worksheet 4).

File Name: Supplementary Data 3

Description: Summary of Candidatus Methanothrix paradoxum metatranscripts based on mappings to
different databases including: mapped metatranscripts to M1 genome mapping (worksheet 1), mapped metatranscripts to a database of isolates and metagenome mcrA (worksheet 2), mapped metatranscripts to the scaffolds in the metagenome (worksheet 3), and the abbreviations and categories of transcripts shown in Supplementary Fig. 8 (worksheet 4).

File Name: Supplementary Data 4

Description: Summary of Candidatus Methanothrix paradoxum sequences (<99% similarity) mined
from public databases (shown in Supplementary 9). Sequence information includes accession numbers and studies (worksheet 1), metadata from each of the studies (worksheet 2), and the distribution of studies by habitat type (worksheet 3).

File Name: Supplementary Data 5

Description: Annotated FASTA of proteins belonging to M1 Candidatus Methanothrix paradoxum M1 genome.

File Name: Supplementary Data 6

Description: Amino acid FASTA of concatenated ribosomal protein alignment from isolate methanogens and Candidatus Methanothrix paradoxum used to construct Supplementary Fig. 4.

File Name: Supplementary Data 7

Description: Amino acid FASTA of S3 ribosomal proteins used to construct Supplementary Fig. 5.

File Name: Supplementary Data 8

Description: Amino acid FASTA of mcrA genes used to construct used to construct Supplementary Fig. 6.

File Name: Supplementary Data 9

Description: Nucleotide FASTA of mcrA genes obtained from wetland metagenomic sequencing and
isolate reference sequences. These sequences served as the database to map metatranscripts to in Fig. 3 and Supplementary Fig. 7.

File Name: Supplementary Data 10

Description: Nucleotide FASTA of Candidatus Methanothrix paradoxum 16S rRNA and near neighbors from environmental datasets used to construct Supplementary Figure 10.
